# Automated digital image quantification of histological staining for the analysis of the trilineage differentiation potential of mesenchymal stem cells

**DOI:** 10.1186/s13287-019-1170-8

**Published:** 2019-02-26

**Authors:** Benjamin Eggerschwiler, Daisy D. Canepa, Hans-Christoph Pape, Elisa A. Casanova, Paolo Cinelli

**Affiliations:** 1 Department of Trauma, University Hospital Zurich, Sternwartstrasse 14, 8091 Zurich, Switzerland; 2 Life Science Zurich Graduate School, University of Zurich, Winterthurerstrasse 190, 8057 Zurich, Switzerland; 3 Center for Applied Biotechnology and Molecular Medicine, University of Zurich, Winterthurerstrasse 190, 8057 Zurich, Switzerland

**Keywords:** Digital image analysis, Dye quantification, Quantification of differentiation potential, Histology, Microscopy, Mesenchymal stem cells, Osteoblasts, Adipocytes, Chondroblasts

## Abstract

**Background:**

Multipotent mesenchymal stem cells (MSCs) have the potential to repair and regenerate damaged tissues and are considered as attractive candidates for the development of cell-based regenerative therapies. Currently, there are more than 200 clinical trials involving the use of MSCs for a wide variety of indications. However, variations in their isolation, expansion, and particularly characterization have made the interpretation of study outcomes or the rigorous assessment of therapeutic efficacy difficult. An unbiased characterization of MSCs is of major importance and essential to guaranty that only the most suitable cells will be used. The development of standardized and reproducible assays to predict MSC potency is therefore mandatory. The currently used quantification methodologies for the determination of the trilineage potential of MSCs are usually based on absorbance measurements which are imprecise and prone to errors. We therefore aimed at developing a methodology first offering a standardized way to objectively quantify the trilineage potential of MSC preparations and second allowing to discriminate functional differences between clonally expanded cell populations.

**Method:**

MSCs originating from several patients were differentiated into osteoblasts, adipocytes, and chondroblasts for 14, 17, and 21 days. Differentiated cells were then stained with the classical dyes: Alizarin Red S for osteoblasts, Oil Red O for adipocytes, and Alcian Blue 8GX for chondroblasts. Quantification of differentiation was then performed with our newly developed digital image analysis (DIA) tool followed by the classical absorbance measurement. The results from the two techniques were then compared.

**Result:**

Quantification based on DIA allowed highly standardized and objective dye quantification with superior sensitivity compared to absorbance measurements. Furthermore, small differences between MSC lines in the differentiation potential were highlighted using DIA whereas no difference was detected using absorbance quantification.

**Conclusion:**

Our approach represents a novel method that simplifies the laboratory procedures not only for the quantification of histological dyes and the degree of differentiation of MSCs, but also due to its color independence, it can be easily adapted for the quantification of a wide range of staining procedures in histology. The method is easily applicable since it is based on open source software and standard light microscopy.

**Electronic supplementary material:**

The online version of this article (10.1186/s13287-019-1170-8) contains supplementary material, which is available to authorized users.

## Background

Quantification of immunohistochemical staining by means of color analysis is a widely employed methodology in research and diagnostics. A major drawback of biochemical assays, such as absorbance measurement, is that it is destructive whereas quantification by image analysis is non-destructive. Furthermore, the possibility to perform automated analysis makes quantification faster, more objective, and less laborious than classical visual examination. Therefore, digital image analysis (DIA) has become a powerful and regularly used tool in a broad range of diagnostic and medical applications [1]. In pathology for example, DIA is used to analyze cell segmentation [2], mitosis [3], or nuclei [4]. Nevertheless, in other fields, this technology is not yet widely used. A possible field of application of DIA is the assessment of trilineage differentiation potential of mesenchymal stem cells (MSCs). Due to their low tumorigenic potential and their ability to rescue apoptotic cells after traumatic exposure, MSCs represent a promising source for regenerative medicine applications [5–8]. They were firstly identified in the bone marrow, but nowadays, they have been isolated and characterized from several adult and fetal tissues [9–12]. The most important challenge for the development of efficacious MSC-based therapies is currently the inability to consistently manufacture homogeneous populations of cells. MSC preparations do not consist of a pure population of cells but are rather a mixture of stem and linage-committed progenitor cells with heterogenous differentiation potential [13, 14]. The gold standard is the assessment of trilineage differentiation potential of MSCs to exhibit adipo-, chondro-, and osteogenesis as a measure of multipotency [15].

Various methods have been developed to characterize the degree of differentiation of MSCs based on transcriptome/proteome quantification. These readouts are very well established and quantitative. However, mRNA or protein levels do not necessarily reflect phenotypic features. The phenotypical characterization of such cell lines is often performed by histological staining [11, 12, 16–18] to prove differentiation down the adipogenic (Oil Red O staining), osteogenic (Alizarin Red S or von Kossa staining), and chondrogenic lineages (Alcian Blue 8GX or Safranin O staining). The main issue with histology is that the readout is binary. Histological staining confirms either the presence or absence of a specific cell type. Often only representative images are shown, lacking the confirmation that bright-field images are an accurate representation of the overall cell monolayer on the culture dish [16, 19–21]. In some cases, quantification of the staining is performed with absorbance measurements [22–25]. However, these protocols are laborious and vulnerable and show low sensitivity and inter-assay reproducibility. The fraction of stained cells is often vanishingly small compared to the total volume of solvent used to elute the dye from the cells. Furthermore, a considerable high amount of unspecifically bound dye is present leading to a low signal to noise ratio.

The development of quantifiable assays able to discriminate functional differences between populations and eventually predict the therapeutic efficacy of MSC products is urgently needed. Therefore, a method with a high signal to noise ratio and high sensitivity to quantify the degree of differentiation on a phenotypic level would be highly beneficial. We therefore aimed at developing a methodology offering first a standardized way to objectively quantify the trilineage potential of MSC preparations and second allowing to discriminate functional differences between clonally expanded cell populations. Moreover, this approach provides easily deployable metrics to assess differentiation efficacy toward osteoblasts, adipocytes, and chondroblasts using specific histological dyes (Alizarin Red S, Oil Red O, and Alcian Blue 8GX).

## Materials and methods

### Gelatine coating

For gelatine coating, 500 μl 0.1% (*w*/*v*) gelatine in ddH_2_O (B. Braun, Germany, Cat. No. 0082479E) was added to each well. After 1 h incubation at room temperature, the gelatine solution was removed and plates were dried at room temperature.

### Cell isolation

Human MSCs were isolated from fat tissue with the consent of the patient according to the Swiss (KEK-ZH: StV 7-2009) and international ethical guidelines (ClinicalTrials.gov Identifier: NCT01218945) as reported previously [26]. The extraction procedure was performed like previously described [27]. MSCs were characterized according to established procedures. Of the 30 isolated primary MSCs, six were selected based on findings in a previous study concerning their differentiation capacity.

### Cell culture

Cells were cultured in DMEM (PAN Biotech, Germany, Cat. No. P04-03550) supplemented with 1% (*v*/*v*) 100× Penicillin-Streptomycin Solution (Biowest, France, Cat. No. L0022), 10% (*v*/*v*) FCS (Biowest, France, Cat. No. S181S), and 1% (*v*/*v*) 200 mM l-glutamine solution (Sigma-Aldrich, USA, Cat. No. G7513), in a humidified atmosphere containing 5% CO_2_. The medium was exchanged every 3–4 days, and cells were split using 1× Trypsin-EDTA (Gibco/Life Technologies, USA, Cat. No. 25200-056) when reached 90–95% confluence.

For osteogenesis, MSCs were seeded on gelatine-coated Nunc™ 24-well plates (Thermo Fisher, USA, Cat. No. 142475) at a density of 1.5 × 10^4^ cells/cm^2^. After 24 h, differentiation was induced using StemPro® Osteogenesis Kit (Gibco/Life Technologies, USA, Cat. No. A10072-01). Cells were regularly checked for morphology, and the medium was exchanged every 3–4 days.

For adipogenesis, MSCs were seeded on Nunc™ 48-well plates (Thermo Fisher, USA, Cat. No. 150687) at a density of 2 × 10^4^ cells/cm^2^. After 24 h, differentiation was induced using the StemPro® Adipogenesis Kit (Gibco/Life Technologies, USA, Cat. No. A10070-01). Cells were regularly checked for morphology, and the medium was exchanged every 3–4 days.

For chondrogenesis, MSCs were seeded on Nunc™ 24-well plates (Thermo Fisher, USA, Cat. No. 142475) at a density of 6 × 10^3^ cells/cm^2^. After 24 h, differentiation was induced with the StemPro® Chondrogenesis Kit (Gibco/Life Technologies, USA, Cat. No. A10071-01). Cells were regularly checked for morphology, and the medium was exchanged every 3–4 days.

### Cell staining

After 14, 17, and 21 days of differentiation, cells were fixed and stained for light microscopy.

Osteoblasts were washed with 1× PBS (Kantonsapotheke Zürich, Switzerland, Cat. No. A171012) and fixed with 4% (*v*/*v*) formaldehyde (Sigma, USA, Cat. No. F8775) in 1× PBS for 30 min. After washing twice with ddH_2_O, Alizarin Red staining solution (0.7 g Alizarin Red S (Sigma, USA, Cat. No. A5533) diluted in 50 ml ddH_2_O at pH = 4.2) was added for 20 min. Afterwards, cells were washed four times with ddH_2_O, dried, and stored in the dark until image acquisition.

Adipocytes were washed with 1× PBS and fixed with 10% (*v*/*v*) formaldehyde in 1× PBS for 65 min. After 5 min, the formaldehyde solution was exchanged. After washing twice with ddH_2_O, wells were rinsed with 60% (*v*/*v*) 2-propanole (Sigma-Aldrich, USA, Cat. No. 59300) in ddH_2_O and dried. Oil Red O solution (0.15 g Oil Red O (Sigma-Aldrich, USA, Cat. No. O0625) diluted in 50 ml 60% (*v*/*v*) 2-propanole in ddH_2_O) was added for 10 min. After four washing steps with ddH_2_O, the plates were dried and image acquisition was performed immediately after.

Chondroblasts were washed with 1× PBS and fixed with 4% (*v*/*v*) formaldehyde in 1× PBS for 20 min. Afterwards, cells were washed twice with ddH_2_O followed by a 3-min incubation at RT with 3% (*v*/*v*) acetic acid (Merck Millipore, Germany, Cat. No. 100063) in ddH_2_O. Alcian Blue solution (0.1 g Alcian Blue 8GX (Sigma, USA, Cat. No. A5268) diluted in 10 ml 3% (*v*/*v*) acetic acid in ddH_2_O at pH = 2.5) was added for 60 min followed by a washing step with 1 M HCl for 3 min. Afterwards, wells were washed four times with ddH_2_O. Multi-well dishes were afterwards stored in the dark until images were acquired.

### Absorbance measurement

Alizarin Red S was eluted from stained osteoblasts with 300 μl 10% (*w*/*v*) cetylpyridinium chloride in an aqueous 0.01 M Na_2_HPO_4_/NaH_2_PO_4_ solution at pH = 7 for 1 h. One hundred fifty microliters was transferred on a 96-well plate, and absorbance was measured at 560 nm. Ten percent (*w*/*v*) cetylpyridinium chloride in an aqueous 0.01 M Na_2_HPO_4_/NaH_2_PO_4_ solution was used as blank.

Oil Red O was eluted from the stained adipocytes with 150 μl 100% 2-propanole. After 1 min, the same amount of ddH_2_O was added and incubated for 4 min. One hundred fifty microliters was pipetted into a 96-well plate, and absorbance was measured at 520 nm. Fifty percent (*v*/*v*) 2-propanole in ddH_2_O was used as blank.

### Image acquisition

Images were acquired with a Cytation 5 imaging reader (BioTek, USA, Cat. No. CYT5MPV). Whole wells were scanned in color bright-field mode with default settings regarding illumination and image capture. Single images were acquired with a horizontal overlap of 200 μm and a vertical overlap of 250 μm. One well on a 24-well plate needed 9 (horizontal) × 13 (vertical) images for stitching. For a 48-well plate, 7 (horizontal) × 10 (vertical) images were required.

### Image processing and analysis

Single images were stitched together using Gen 5 image prime software (BioTek, USA, V3.03). Since the Cytation 5 imaging reader creates for each basic color (red, green, blue) a separate 16-bit image, a composite file was created using the open source software Cell Profiler [28]. Image analysis was performed with the open source software Fiji [29]. For any mathematical operation during the analysis steps, images were converted into a 32-bit floating point format to avoid pixel saturation. A macro was written in ImageJ macro language to analyze stitched images in batch mode. Analyzed parameters included area and area fraction.

### Quantification of lineage-specific differentiation potential

For DIA, the measured values after 14, 17, and 21 days of differentiation were added up and then divided by 1000. For absorbance indeed, the measured values were added up and multiplied by 1000. This value is termed “Ranking Points”.

### Statistical analysis

All data analysis was carried out by using Graph Pad Prism 8 software (GraphPad Software, USA, Version 8.0.0). Unless otherwise stated, *p* values were calculated using Student’s *t* tests and error bars indicate the standard deviation. Significance levels were subdivided into *(*p* value ≤ 0.05), **(*p* value ≤ 0.01), ***(*p* value ≤ 0.001), and ****(*p* value ≤ 0.0001). For correlation analysis, Pearson’s *r* value was calculated.

## Results

### Image acquisition and analysis protocol

**Fig. 1:**
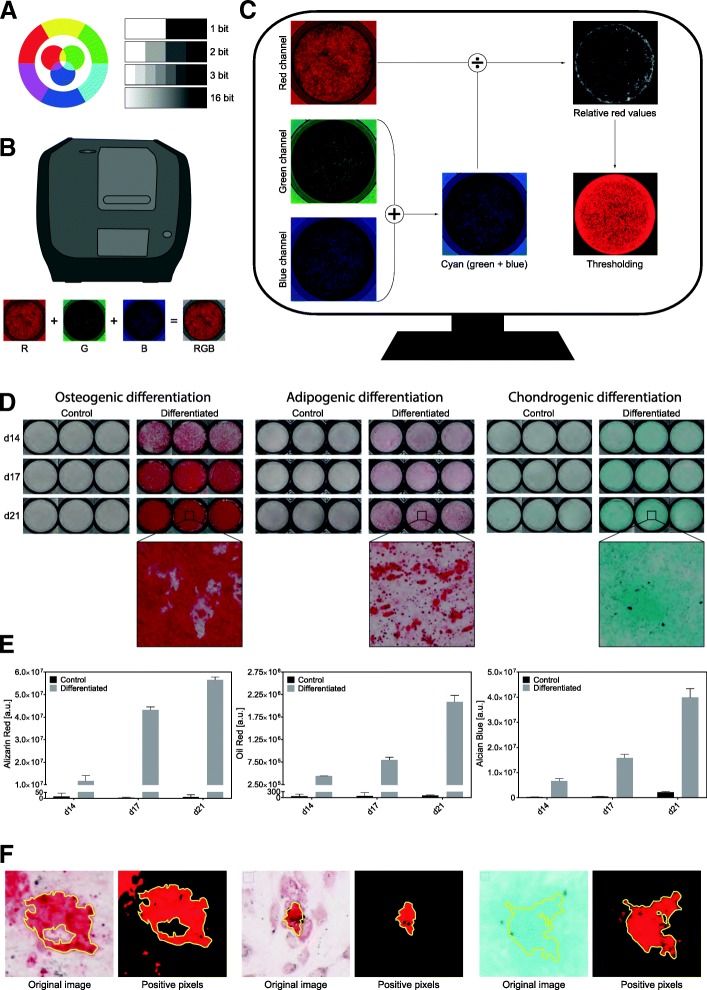
Overview of image acquisition and processing. Digital images are composed of the three basic colors red, green, and blue (**a**). The observed color is determined by the bit depth of each of the three basic colors. In a first step, images were acquired for each basic color separately (**b**), followed by an image processing step (**c**) where the pixel values of the color of interest was divided by its complementary color. In our case, the red channel was divided by cyan, a mixture of green and blue. The thresholding on the bit depth was performed after this step. MSCs differentiated for 14, 17, and 21 days into osteoblasts, adipocytes, and chondroblasts were quantified (*n* = 3) by scanning the whole well of multi-well plates (**d**). DIA quantification of the staining showed a constant increase in differentiation for all three lineages (*n* = 3, error bars represent SD) (**e**). The system is very robust. Most of the background as well as other disturbing factors, such as dirt, are eliminated (**f**)

In a first step, an image acquisition protocol was established to ensure consistent data generation. All relevant parameters, such as exposure time, white balance, light intensity, and plate dimensions, were fixed for all image acquisitions. The basic principle behind our method is the fact that a dye absorbs its complementary color leading to a specific bit depth value for each pixel (Fig. 1a). Our system is using a 16-bit CCD camera (2^16^ bit depth increments), and samples are illuminated with three different LEDs with tight emission spectra and emission maxima at 460 nm (blue light), 523 nm (green light), and 623 nm (red light) to generate a color bright-field image (Fig. 1b). Because the relationship between spectral absorbance and stain concentration is only linear under monochromatic conditions, we can assume for our purposes that light transmission is linear with the pixel values in each channel [30, 31]. Therefore, the relative intensity of the histological dye is analyzed by dividing the signal of the dye by the signal of its complementary color. This means that for the red staining Alizarin Red S and Oil Red O, the red channel is divided by cyan, a mixture of green and blue, which represents the complementary color of red (Fig. 1c). A signal with a ratio of $\frac{\mathrm{red}\ \mathrm{channel}}{\mathrm{green}\ \mathrm{channel}+\mathrm{blue}\ \mathrm{channel}}\ge 1$ was considered as a positive signal because the signal from the red channel is at least twice as strong as the average signal from the green and the blue channel. All pixels within the region of interest (ROI), which represents the bottom of the well with the cell monolayer, fulfilling this ratio were counted. Alizarin Red S and Oil Red O staining showed a very strong signal in the red channel. For Alizarin Red S, as an example, a mean pixel value of 34,437 was initially measured, indicating a low absorbance of the red light fraction. For the green channel, a mean pixel value of 4521 was determined and for the blue channel a mean pixel value of 5368. This indicates that most of the green and the blue light fraction is absorbed by the dye, leading to the red color perception. Because of the dominance of the red channel values, the green and the blue channel were neglected for the signal and the formula to define a positive pixel was maximally simplified. Oil Red O staining showed similar values. For Alcian Blue 8GX, which was used to confirm the presence of chondroblasts, the signal is found in the green and the blue channel and the corresponding complementary color, which is absorbed, is red. Therefore, a ratio of $\frac{\mathrm{green}\ \mathrm{channel}+\mathrm{blue}\ \mathrm{channel}}{\mathrm{red}\ \mathrm{channel}}\ge 4$ represents a positive signal since the average signal intensity in the green and the blue channel is twice as high as in the signal in the red channel. Initial measurements of Alcian Blue 8GX stainings revealed that the green and the blue channel have similar mean pixel values (32,622 and 33,774) which are multiple times larger than the mean values from the red channel (9362). These values underline that mainly the red component of light is absorbed. Thus, the contribution of the red channel to the final signal was neglected, and the formula was again simplified as previously done for Alizarin Red S and Oil Red O quantification.

### Testing of the digital image analysis method

To test our approach, MSCs, isolated from the stromal vascular fraction of fat tissue from six healthy individuals that underwent adipose tissue excision, were differentiated into osteoblasts, adipocytes, and chondroblasts. After 14, 17, and 21 days of differentiation, cells were fixed and stained either with Alizarin Red S, Oil Red O, or Alcian Blue 8GX to confirm the corresponding cell lineage. Cells were cultured either in differentiation medium or in DMEM (control). Images of the entire well for each condition were taken for analysis (Fig. 1d). When we applied our quantification protocol (Additional file 1: Figure S1) to evaluate the differentiation in the MSC lines, we confirmed that the new DIA approach was able to reliably detect different color stains (Fig. 1e and Additional file 2: Figure S2). Moreover, independently from the dye used, the signal was nicely separated between differentiated and control cells and nearly all background signal was eliminated. (Fig. 1e). We were also able to detect changes during the differentiation into each of the three lineages and to precisely monitor the differentiation progress of MSCs over time (Fig. 1e).

Furthermore, with a × 40 magnification, our DIA approach allowed to analyze the histological staining on an extremely high resolution and the thresholding was highly effective. Noise was massively reduced and the whole system was resilient against external disturbing factors such as dirt. This was true for all the three staining types: Alizarin Red S, Oil Red O, and Alcian Blue 8GX (Fig. 1f).

### System validation—comparison with standard absorbance measurements

**Fig. 2:**
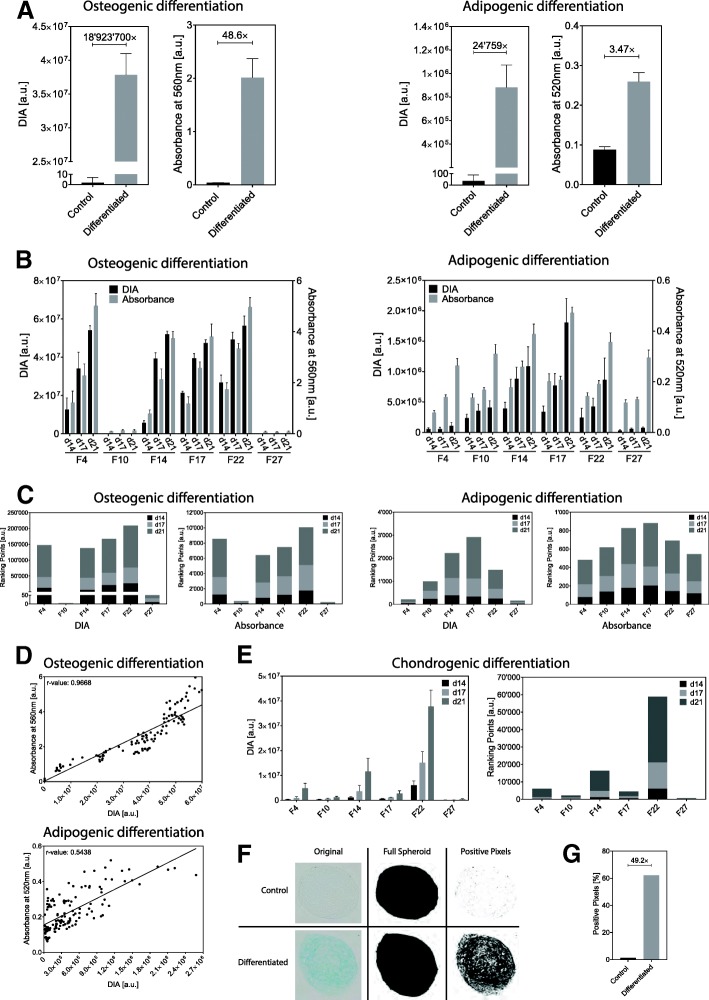
Validation of the DIA approach. The DIA approach was compared to absorbance measurements and showed an increased signal to noise ratio for both osteogenic and adipogenic differentiation (**a**). (*n* = 18 for osteogenic differentiation, *n* = 9 for adipogenic differentiation, error bars represent SD). Six different cell lines were differentiated into osteoblasts and adipocytes and afterwards analyzed with DIA and absorbance measurements (**b**). (*n* = 9 for all cell lines except for F22 *n* = 3, error bars represent SD). The ranking of all six cell lines showed a signal overestimation for absorbance measurements and a superior sensitivity of the DIA approach (**c**). Correlation analysis for osteogenesis revealed that DIA does reliably quantify Alizarin Red S. The correlation between absorbance measurements and DIA for adipocytes is less strong (**d**). The reason for this is a signal overestimation originating from absorbance measurements for weakly differentiated cells (e.g., controls). DIA also allows to quantify histological dyes that cannot be quantified by absorbance (**e**). Chondrogenic differentiation was quantified with DIA using Alcian Blue 8GX-stained cells (*n* = 9, error bars represent SD). Chondroblasts that were differentiated in 3D were cut into multiple cross sections, and positive pixels were compared to the total number of pixels of the spheroid (**f**) leading to a quantitative expression of the chondrogenic differentiation (**g**)

The current standard to quantify histological dyes is absorbance measurement. The dye needs to be eluted/extracted from the tissue, and then absorbance is measured at a dye-specific wavelength. In order to validate our system, we again differentiated MSCs into osteoblasts and adipocytes and stained the cells with the corresponding dye after 14, 17, and 21 days of differentiation. The same plate was first subjected to DIA quantification, and thereafter the dye was extracted for absorbance measurements. Comparison of the two methods showed that DIA was more sensitive and eliminated most of the background signal (Fig. 2a). Cell line F14, as an example, that underwent osteogenic differentiation for 17 days showed a signal to noise ratio of nearly 19,000,000× with DIA, whereas with absorbance it was only 48.6× (Fig. 2a). Same applied for adipogenic differentiation. The background was almost completely eliminated with DIA leading to a signal to noise ratio of roughly 25,000× compared to a ratio of 3.47× from absorbance measurement (Fig. 2a).

Next to the DIA protocol with increased sensitivity, we established a system that allows us to rank different mesenchymal stem cell lines according to their differentiation potential toward a distinct lineage. For therapeutic applications of MSCs, it is important to quantify the differentiation potential into the lineage of interest. For this purpose, we differentiated six MSC lines into osteoblasts and adipocytes (Fig. 2b). By adding up the measured values after 14, 17, and 21 days of differentiation, the differentiation potential for each cell line was expressed in a quantitative way and a classification of the lines was therefore possible (Fig. 2c). An ideal cell line produces a large amount of differentiated tissue in a short time period. Hence, our approach takes a time and a quantity component into consideration.

For osteogenic differentiation, it is clear that the line F10 and F27 have a poor ability to generate osteoblasts (Fig. 2b). Nevertheless, with our ranking approach, it is possible to observe that the line F27 differentiated better than the line F10 (Fig. 2c). These slight differences were not observed when absorbance was measured (Fig. 2c), thus confirming the superior sensitivity of the DIA approach compared to the classical absorbance approach. All other cell lines differentiated better and showed a similar differentiation pattern with DIA as well as with absorbance (Fig. 2c). Thus, the use of the highly sensitive DIA method in combination with our quantification procedure allows to highlight the slightest differences with much more precision between osteogenic differentiating cell lines with very low signals.

Adipogenic differentiation was easily measured and quantified with DIA whereas absorbance measurements were extremely difficult, laborious, and vulnerable (Fig. 2b and Fig. 2c). Because Oil Red O staining is eluted with pure 2-propanole, a highly volatile solvent, measured absorbance values may vary massively. Already a slight difference in time between elution and absorbance measurement or variation in room temperature may lead to differences in evaporation and therefore differences in absorbance. Another drawback of Oil Red O absorbance measurement is the residual dye on the sidewall of the wells with cells that underwent differentiation. This residual dye is obviously also eluted leading to false results. Already the smallest amounts lead to unwanted signals in absorbance measurements and therefore compromise the detection limit and lead to a signal overestimation. Especially, cell lines that differentiate in a poor way, such as cell lines F4, F10, and F27, show heavily overestimated absorbance values since the true values are “covered” with the signal from the residual dye on the sidewalls (Fig. 2b and Additional file 3: Figure S3). Only for cell lines that differentiated well into adipocytes, the noise became negligible (Fig. 2b). Furthermore, absorbance measurements lead to wrong conclusions regarding the adipogenic differentiation potential (Fig. 2c). Thus, especially for adipogenic differentiation potential measurements, a highly sensitive method like DIA is of extreme importance, since the dye elution step, which is the one causing the measurement’s variability, is completely omitted.

Correlation analysis between DIA and absorbance values revealed a very good correlation (Pearson’s *r* = 0.9668) between DIA and absorbance measurement for Alizarin Red S quantification and a poor correlation (Pearson’s *r* = 0.5438) for Oil Red O (Fig. 2d).

The weak correlation of Oil Red O is caused by the very limited detection capabilities of absorbance measurements which originate from the residual dye on the sidewalls. In our experiments, approximately 22,400 MSCs were exposed to adipogenic medium per well. Even if all cells differentiated into adipocytes, the total volume of Oil Red-stained lipids would still be extremely small compared to the total volume. The final volume during the elution process was 300 μl. It is known that, on average, a single adipocyte has a volume of 0.0003 μl, leading to a total volume of 6.72 μl Oil Red O-stained lipids if all cells are differentiated into adipocytes [32]. These low volumes make absorbance measurements extremely susceptible to noise from unspecifically bound Oil Red O stain.

Osteogenic differentiation is less affected. Osteoblasts produce larger amounts of extracellular matrix which are stained by Alizarin Red S. Therefore, the residual dye on the sidewalls of each well is less disturbing.

Another advantage of the DIA method is its ability to quantify histological dyes that cannot be brought into a solution. Alcian blue is an example for such a dye. It is often used to show the presence of cartilage by staining the acidic carbohydrates which occur in the cartilage matrix. However, during standard staining protocols where Alcian Blue 8GX-stained samples are washed with water, Alcian Blue 8GX forms an insoluble phthalocyanine complex [33]. This is only soluble in highly concentrated sulfuric acid, which destroys the complex and therewith the chromatic properties of the dye. Therefore, no reliable protocol was established to measure Alcian Blue 8GX-stained cartilage from cell culture or tissue samples by absorbance spectroscopy. However, with our DIA approach, we were able to quantify the chondrogenic differentiation potential of six different MSC lines and found F22 to have the highest and F27 the lowest chondrogenic differentiation potential (Fig. 2e).

A major advantage of our DIA approach is its flexibility. It is color independent and not only applicable to cell monolayers in multi-well plates. As a proof of principle, chondroblasts grown in spheroids were also quantitatively assessed. Since all spheroids vary in their size, quantification via DIA was adjusted and the number of positive pixels was determined compared to the total number of pixels of the spheroid. Therefore, multiple cross sections of a spheroid that was grown in a chondrogenic medium were stained with Alcian Blue 8GX followed by image acquisition and analysis (Fig. 2f, g).

## Discussion

Over the past years with the increase of computational power, there has been significant progress in image analysis [34, 35]. Similarly, the mesenchymal stem cell research field expanded in the last years exponentially due to the unique therapeutic properties of these cells. Nevertheless, although the excitement on the potential use of MSCs in regenerative medicine is very big, the development of robust techniques for their unbiased characterization and classification is still very poor. We describe here the principle and the validation of an image analysis approach that simplifies the laboratory procedures to objectively quantify and classify the degree of differentiation as well as the differentiation potential among different MSC cell lines or cell subpopulations. This approach is of course not limited to MSCs only but can be applied to any type of cells and tissue and also staining dye.

The method proposed here produces robust results with a superior sensitivity compared to absorbance measurements. In fact, very low signals, such as Oil Red O-stained poorly differentiated adipocytes, could be detected and analyzed with our approach whereas absorbance measurement was only reliable for large values of very well-differentiated cell lines.

Furthermore, absorbance measurements are prone to errors, and the sample preparation procedures are time-consuming and difficult to standardize. Since Oil Red O is eluted with pure 2-propanole, a highly volatile solvent, a lot of variability is added to the absorbance measurement and makes the whole measurement highly vulnerable to noise. All Oil Red O and Alizarin Red S stainings of differentiated cells lead to residual dye on the wall of a well, lowering the sensitivity and signal to noise ratio when quantified by absorbance measurements. With our approach, we completely eliminate the distaining step, avoiding the introduction of additional noise to the measurements.

On the other hand, variance is increased in DIA compared to absorbance measurements. Due to the high sensitivity, little differences which are not detected by absorbance measurements may lead to a considerable signal difference during DIA quantification.

Compared to other image analysis procedures, such as color deconvolution, our system has a superior sensitivity. Color deconvolution is based on 8-bit RGB images and needs a preliminary definition of the color deconvolution vectors. This definition is difficult and therefore prone to errors. Our bit-depth-independent DIA approach works with 16-bit images which represents a 256× fold increase in sensitivity/resolution compared to 8-bit images, and the difficult vector definition step is omitted. Moreover, color deconvolution is limited to stoichiometric staining reactions [36]. Thus, color deconvolution is not suitable to quantify immunohistochemical stainings. Our approach also allows to quantify such stainings because we only consider pixels above or below a predefined threshold either as positive or negative. We do not quantify the spatial amount of staining as it is done during color deconvolution.

## Conclusion

The new proposed method clearly proved its superiority compared to the conventionally used methods for MSC differentiation, quantification, and classification, and it represents therefore for the MSC research field a very important new tool.

Moreover, a major advantage of this method is its flexibility: Neither it is limited to a certain dye or cell type nor to cell monolayers in multi-well plates, and by using *Fiji’s* batch mode, it is possible to automatically analyze large numbers of sections. Data credibility is also increased with availability of the original images.

In conclusion, with its high sensitivity, our approach can be used as a diagnostic tool because it allows to quantitatively distinguish cell lines and their differentiation capability based on their phenotype.

## Additional files

Additional file 1: **Figure S1.** Workflow for DIA. The flow chart shows all crucial steps for proper image analysis/quantification. Each step is described in the “Materials and methods” section in detail. (PDF 296 kb)

Additional file 2: **Figure S2.** Illustration of original and thresholded images. In this graph, original images are shown together with its corresponding thresholded image for all three histological dyes used, Alizarin Red S for osteoblasts, Oil Red O for adipocytes, and Alcian Blue 8GX for chondroblasts. The yellow circle highlights the ROI in which the pixels were analyzed. (PDF 493 kb)

Additional file 3: **Figure S3.** Illustration of superiority of the DIA approach. The images illustrate the differentiation into adipocytes after 14, 17, and 21 days of differentiation. Cell line F14 represents a cell line with a high adipogenic potential whereas F27 represents a cell line with a low adipogenic potential. The comparison between DIA and absorbance measurements reveals that absorbance measurements overestimate low signals. According to absorbance measurements, the degree of differentiation of cell line F27 after 21 days is roughly the same as for cell line F14 after 17 days of differentiation. Absorbance measurements are extremely prone to errors because a lot of unspecifically bound dye (especially from the side walls of a well) is brought into the solution. (PDF 488 kb)

## Abbreviations

CCD: Charge coupled device
DIA: Digital image analysis
MSC: Mesenchymal stem cells
RGB: 3 channels (red, green, blue)
ROI: Region of interest
