# Supplementary figures and images for: Automated digital image quantification of histological staining for the analysis of the trilineage differentiation potential of mesenchymal stem cells
Source: Stem Cell Res Ther. 2019 Feb 26;10:69. doi: 10.1186/s13287-019-1170-8 (PMC6390603; doi:10.1186/s13287-019-1170-8)

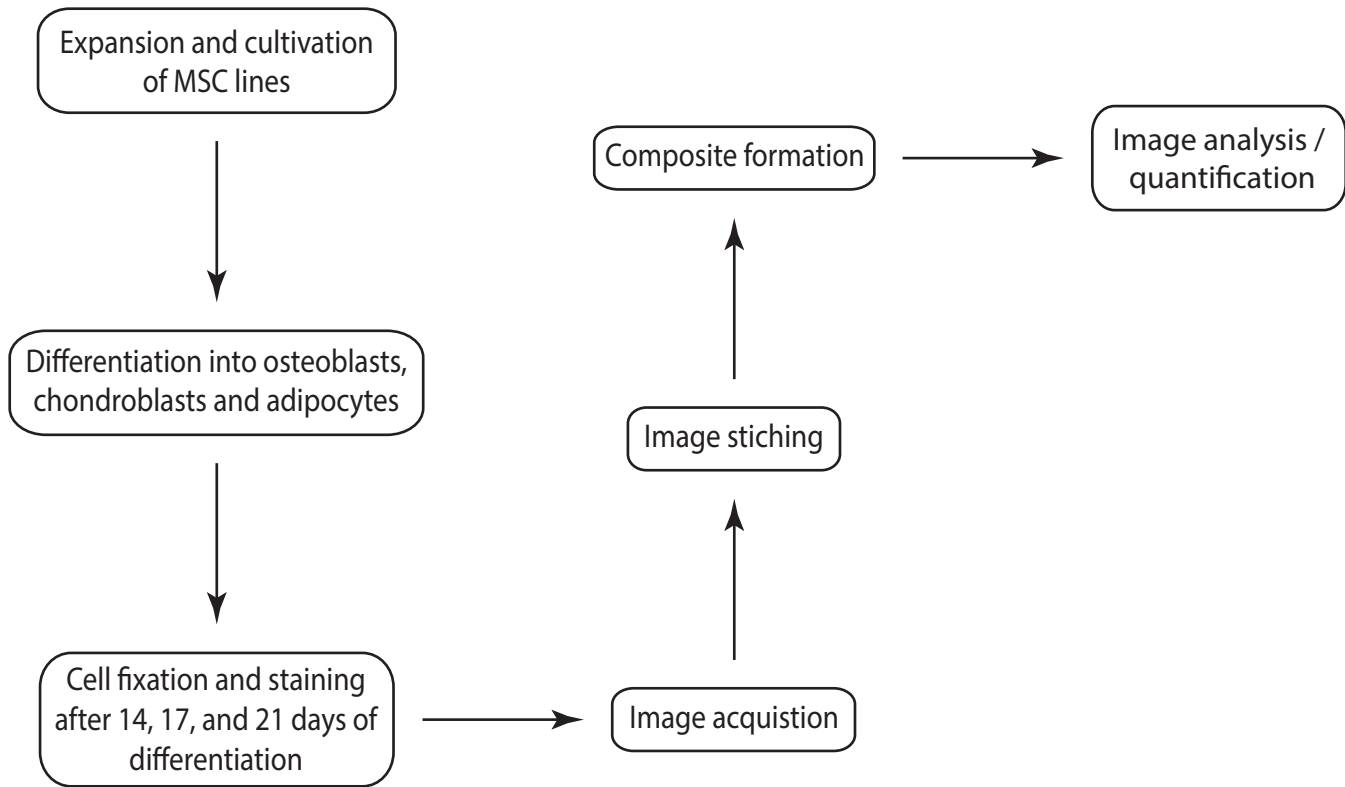

Supplement: Supplementary file 1 — Figure S1. Workflow for DIA. The flow chart shows all crucial steps for proper image analysis/quantification. Each step is described in the “Materials and methods” section in detail. (PDF 296 kb) [file 13287_2019_1170_MOESM1_ESM.pdf]

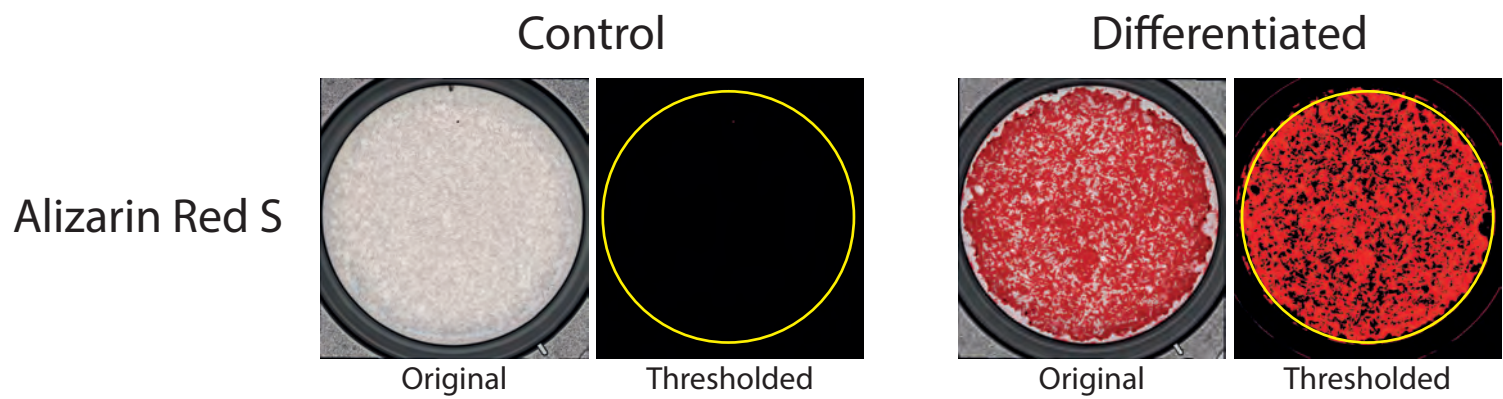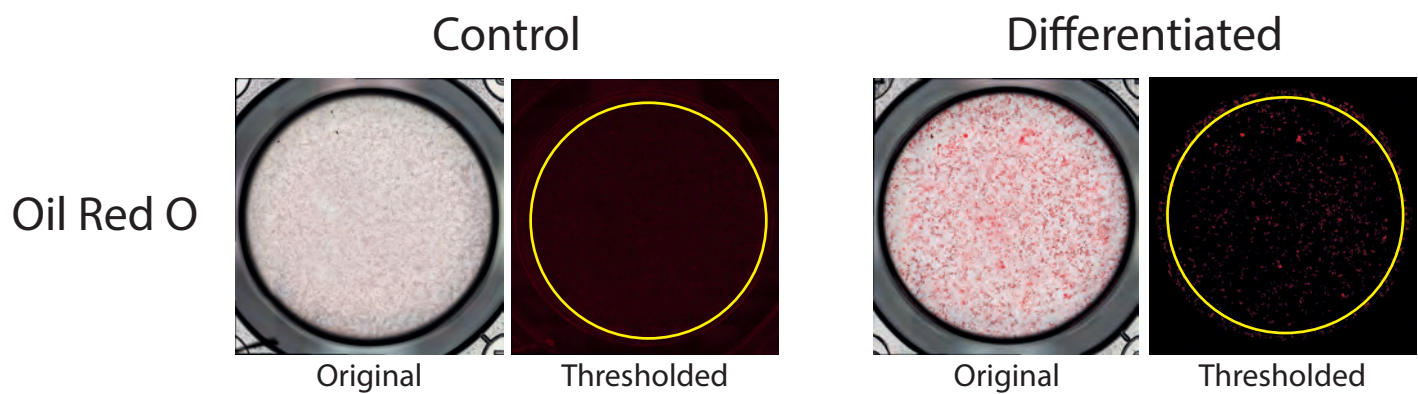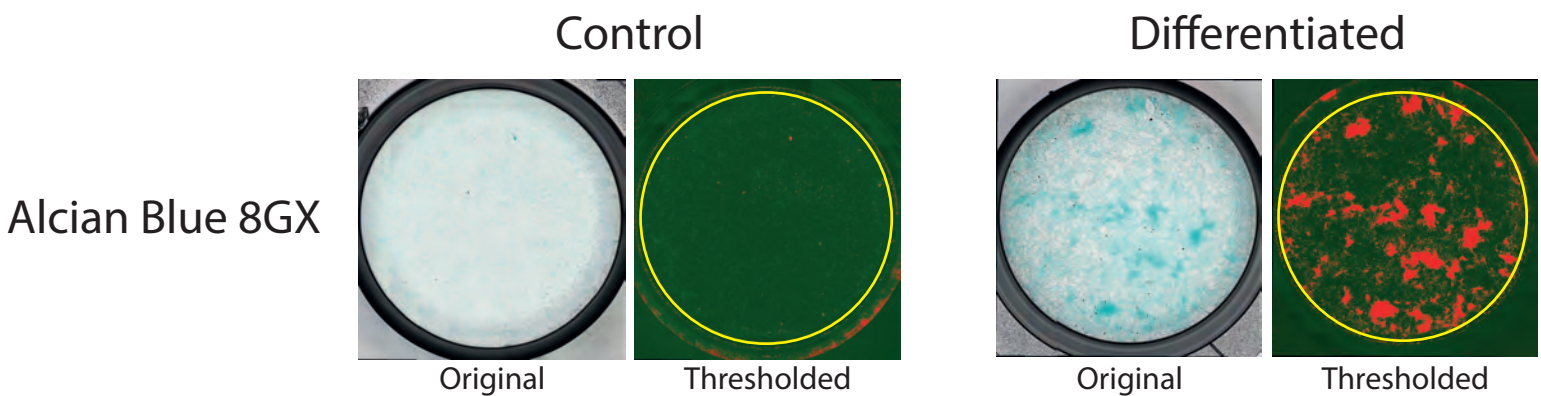

Supplement: Supplementary file 2 — Figure S2. Illustration of original and thresholded images. In this graph, original images are shown together with its corresponding thresholded image for all three histological dyes used, Alizarin Red S for osteoblasts, Oil Red O for adipocytes, and Alcian Blue 8GX for chondroblasts. The yellow circle highlights the ROI in which the pixels were analyzed. (PDF 493 kb) [file 13287_2019_1170_MOESM2_ESM.pdf]
