# Supplementary material for: Automated digital image quantification of histological staining for the analysis of the trilineage differentiation potential of mesenchymal stem cells
Source: Stem Cell Res Ther. 2019 Feb 26;10:69. doi: 10.1186/s13287-019-1170-8 (PMC6390603; doi:10.1186/s13287-019-1170-8)

Adipogenic differentiation

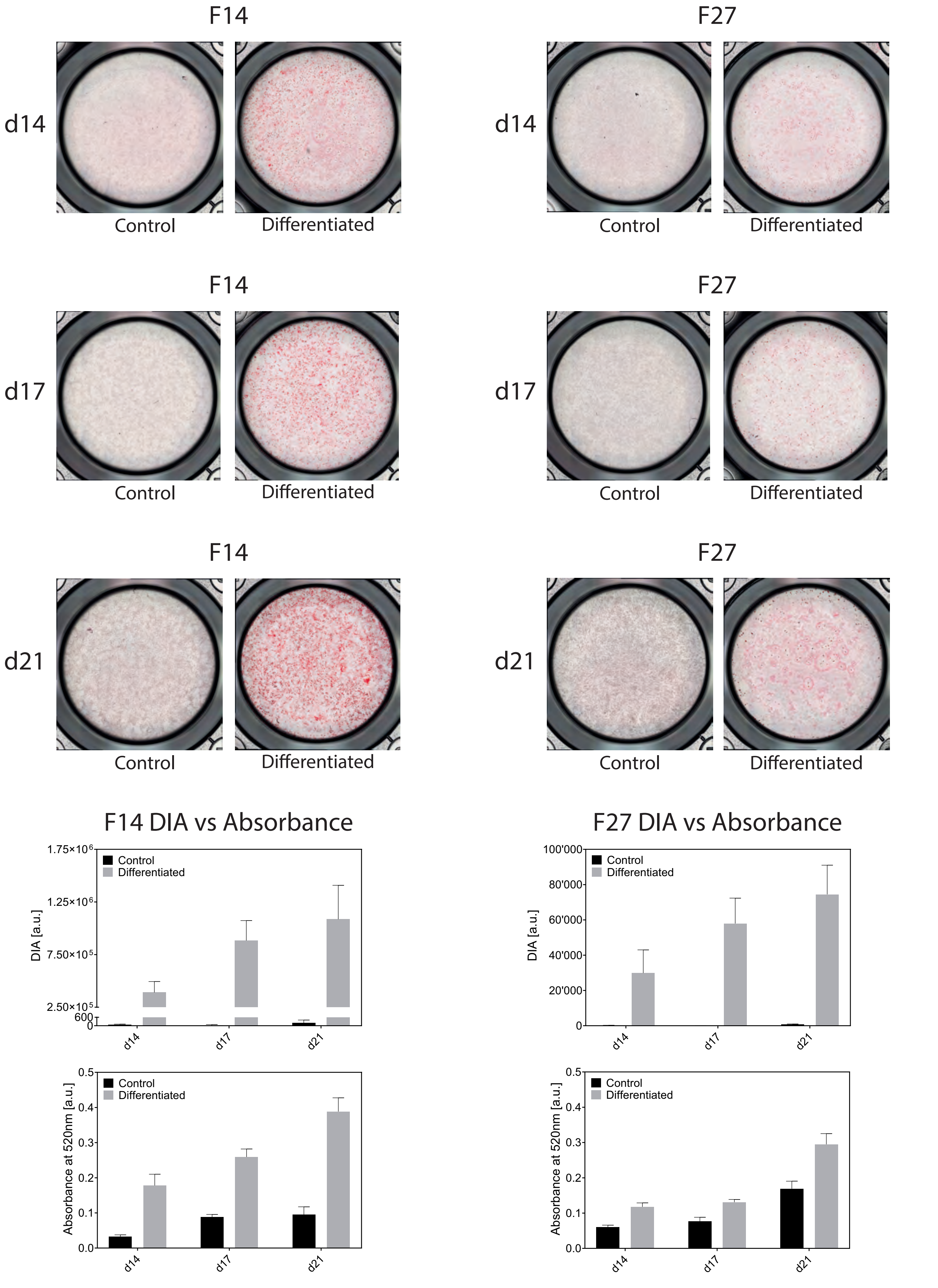

Supplement: Supplementary file 3 — Figure S3. Illustration of superiority of the DIA approach. The images illustrate the differentiation into adipocytes after 14, 17, and 21 days of differentiation. Cell line F14 represents a cell line with a high adipogenic potential whereas F27 represents a cell line with a low adipogenic potential. The comparison between DIA and absorbance measurements reveals that absorbance measurements overestimate low signals. According to absorbance measurements, the degree of differentiation of cell line F27 after 21 days is roughly the same as for cell line F14 after 17 days of differentiation. Absorbance measurements are extremely prone to errors because a lot of unspecifically bound dye (especially from the side walls of a well) is brought into the solution. (PDF 488 kb) [file 13287_2019_1170_MOESM3_ESM.pdf]
